# Perioperative Care of Cancer Patients Treated with Immune Checkpoint Inhibitors: Current Evidence and Clinical Considerations—A Scoping Review

**DOI:** 10.3390/cancers18162654

**Published:** 2026-08-17

**Authors:** Ioana Roxana Codru, Liliana Vecerzan

**Affiliations:** 1 Faculty of Medicine, Lucian Blaga University of Sibiu, 550169 Sibiu, Romania; ioanaroxana.bera@ulbsibiu.ro; 2 Anesthesia and Intensive Care Department, Clinical Emergency Hospital of Sibiu, 550245 Sibiu, Romania; 3 Oncology Department, Clinical Military Emergency Hospital of Sibiu, 550024 Sibiu, Romania

**Keywords:** immune checkpoint inhibitors, perioperative immunotherapy, cancer surgery, immune-related adverse events, perioperative management, surgical oncology, neoadjuvant immunotherapy, adjuvant immunotherapy, perioperative care

## Abstract

**Background**: Immune checkpoint inhibitors (ICIs) have moved from the metastatic setting into neoadjuvant, adjuvant, and fully perioperative strategies across several solid tumors. This shift has created a new clinical interface between medical oncology, surgery, anesthesia, pathology and postoperative care because immune activation may improve pathological response and survival while also generating immune-related adverse events (irAEs) that mimic or aggravate perioperative complications. **Methods**: We conducted a scoping review according to PRISMA-ScR. PubMed/MEDLINE and Web of Science were searched for studies published between 2016 and 2026 that evaluated adult patients with solid tumors receiving ICIs in relation to surgery. Thirty-three studies were included and synthesized descriptively across surgical feasibility, perioperative safety, irAEs, anesthetic considerations, and oncological outcomes. **Results**: The strongest evidence was found in resectable non-small-cell lung cancer, where neoadjuvant or perioperative ICI-based regimens improved pathological response and, in several trials, event-free or overall survival. Evidence in triple-negative breast, bladder, gastric/gastroesophageal junction, and ovarian cancers supported broader applicability but remained heterogeneous, with variable efficacy across tumor types and treatment regimens. Surgery following ICI exposure was generally feasible, without a consistent increase in postoperative mortality. However, pneumonitis, myocarditis, endocrinopathies, hepatitis, colitis, and cytokine release syndrome may mimic conventional postoperative complications. Direct evidence comparing anesthetic or perioperative management strategies was scarce. **Conclusions**: Perioperative ICI-based therapy is no longer an experimental concept, but its safe implementation requires structured preoperative screening, individualized surgical timing, organ-specific toxicity surveillance, careful corticosteroid decision-making, and close multidisciplinary communication. Future prospective studies should integrate standardized perioperative endpoints, anesthesia-related variables, biomarker-driven risk stratification, and long-term oncological outcomes.

## Simple Summary

Immune checkpoint inhibitors (ICIs) have become an integral component of perioperative treatment for several solid tumors, particularly resectable non-small cell lung cancer. Current evidence indicates that surgery following ICI therapy is generally feasible and safe, without a consistent increase in postoperative morbidity or mortality. However, immune-related adverse events may mimic common perioperative complications, requiring careful differential diagnosis and multidisciplinary management. Thorough preoperative assessment, individualized surgical timing, and vigilant postoperative monitoring are essential to optimize patient outcomes. Although oncologic benefits are well established in selected malignancies, important knowledge gaps remain regarding optimal anesthetic strategies, perioperative management, and long-term safety. Further prospective multidisciplinary studies are needed to establish standardized perioperative care pathways for patients receiving immunotherapy.

## 1. Introduction

Immune checkpoint inhibitors (ICIs) have reshaped the therapeutic architecture of solid tumors, initially in advanced disease and, more recently, in treatment pathways designed with curative intent. Their movement into the perioperative setting represents more than a simple temporal shift of systemic therapy before or after surgery. It reflects a change in biological strategy: the tumor is no longer regarded only as a mass to be resected but also as an antigenic field capable of priming or amplifying systemic antitumor immunity before definitive local treatment. In early-stage non-small-cell lung cancer (NSCLC), contemporary evidence has rapidly evolved from biological rationale and phase II feasibility studies to randomized perioperative trials, establishing this disease as the model through which many principles of perioperative immuno-oncology are now being defined [1,2,3,4,5,6].

The principal FDA-approved ICIs used in clinical practice include PD-1 inhibitors (nivolumab, pembrolizumab, and cemiplimab), PD-L1 inhibitors (atezolizumab, durvalumab, and avelumab), and the CTLA-4 inhibitor ipilimumab. These agents restore antitumor immune activity by disrupting inhibitory signaling pathways that normally limit T-cell activation [7].

In this context, neoadjuvant immunotherapy is ICI treatment given before surgery, while adjuvant immunotherapy starts after surgical resection. A comprehensive perioperative approach combines preoperative treatment with planned postoperative continuation. In this review, the term “perioperative care” broadly refers to managing patients receiving ICIs before, during, or after surgery, especially when such exposure might affect surgical timing, anesthetic assessment, postoperative complications, or recovery. Although these treatment settings are connected, they should not be viewed as interchangeable.

The perioperative period presents a promising opportunity for immunotherapy, as it focuses on three key goals: eliminating small metastatic disease, enhancing surgical outcomes, and ensuring complete tumor removal. Unlike treating advanced metastatic cancer, where the immune system may be worn out, using immune checkpoint inhibitors (ICIs) before surgery can harness the body’s natural defenses while the tumor is still intact. However, this timing is critical; excessive side effects, delayed recovery, or immune-related complications can jeopardize the chance for a cure. Therefore, it is essential to assess perioperative immunotherapy not only by cancer outcomes but also by surgical feasibility, recovery challenges, coordination among healthcare providers, and long-term patient survival [1,2,4,5,6,8,9].

The strongest body of evidence comes from resectable NSCLC. The Current Oncology review by Lazzari and colleagues summarized the expanding clinical trial landscape and emphasized both the biological rationale and the need for predictive biomarkers in stage I-III NSCLC [1]. SAKK 16/14 subsequently provided an important phase II signal that durvalumab added to neoadjuvant chemotherapy, followed by surgery and postoperative durvalumab, was feasible and safe in patients with resectable stage IIIA(N2) disease, exceeding historical expectations for this high-risk population [2]. The long-term translational update from Schmid and colleagues deepened this evidence by linking perioperative anti-PD-L1 therapy and chemotherapy to durable clinical outcomes, while also showing that immune dynamics and tumour immune architecture may help explain why some patients achieve durable benefit whereas others relapse despite apparently adequate multimodality therapy [3].

Randomized phase III data have now strengthened the clinical foundation of perioperative ICI use in NSCLC. KEYNOTE-671 showed that neoadjuvant pembrolizumab plus platinum-based chemotherapy followed by adjuvant pembrolizumab improved clinically meaningful outcomes compared with neoadjuvant chemotherapy alone in patients with resectable early-stage NSCLC, including survival endpoints and pathological response measures [5]. Similarly, the final analysis of RATIONALE-315 reported a benefit of perioperative tislelizumab combined with neoadjuvant chemotherapy in resectable NSCLC, adding to the growing perioperative evidence base [6]. These studies are important because they move perioperative immunotherapy beyond response enthusiasm into a more mature therapeutic framework, where efficacy must be read alongside safety, resection rates, postoperative outcomes, and the burden of treatment before and after surgery.

Beyond single-agent PD-1 or PD-L1 blockade combined with chemotherapy, newer approaches are testing whether dual checkpoint inhibition can intensify immune priming without sacrificing surgical safety. NEOpredict-Lung, evaluating short-course preoperative nivolumab with or without the LAG-3 inhibitor relatlimab, reported encouraging feasibility and long-term outcomes in resectable NSCLC [4]. Although such phase II data require cautious interpretation, they highlight a central question for perioperative care: how much immune activation is sufficient before surgery, and at what point does additional immune modulation add complexity without proportional clinical benefit? This question is not merely oncological; it is operational and surgical. The optimal neoadjuvant ICI regimen must be effective, but also predictable, time-limited, and compatible with a safe path to resection.

The perioperative ICI paradigm is not confined to lung cancer. In early-stage triple-negative breast cancer (TNBC), the KEYNOTE-522 platform has established the concept of neoadjuvant pembrolizumab plus chemotherapy followed by adjuvant pembrolizumab; the Japan subgroup analysis further supported the consistency of this approach in a defined population of patients with high-risk early TNBC [10]. In muscle-invasive bladder cancer, the phase II study by Rose and colleagues evaluated gemcitabine and split-dose cisplatin plus pembrolizumab before radical cystectomy and met its prespecified endpoint for pathological downstaging, while being generally feasible in the neoadjuvant setting [11]. These tumor-specific examples demonstrate that perioperative immunotherapy must be interpreted through the biology of each cancer type, the surgical procedure involved, and the tolerance of combining ICI with chemotherapy in patients who still need to undergo definitive local therapy.

In upper gastrointestinal malignancies and ovarian cancer, the evidence illustrates both the promise and the limits of extrapolation. The MONEO study examined perioperative avelumab plus chemotherapy in resectable gastric or gastroesophageal junction adenocarcinoma and reported safe but relatively modest antitumor activity overall, with stronger signals in PD-L1 combined positive score-high tumors [12]. In newly diagnosed stage III or IV ovarian cancer, IMagyn050 tested atezolizumab with bevacizumab and chemotherapy in a randomized phase III design, illustrating the complexity of integrating immunotherapy into regimens already shaped by cytoreductive surgery, platinum-taxane chemotherapy and anti-angiogenic treatment [13]. Together, these data caution against a universal perioperative immunotherapy narrative. The immune contexture, chemotherapy backbone, surgical morbidity, biomarker landscape, and timing of systemic therapy differ profoundly across tumor types.

For the clinician, perioperative immunotherapy creates a new category of risk: toxicity that may masquerade as ordinary perioperative disease. Pneumonitis may be misread as infection, postoperative atelectasis, or pulmonary embolism; colitis may be mistaken for antibiotic-associated diarrhea or surgical complications; hepatitis may be attributed to anesthesia, hepatic congestion, or metastases; and endocrinopathies may present with fatigue, hypotension, hyponatremia, or delayed recovery. Such events are not theoretical. They are clinically relevant because the perioperative interval is already crowded with inflammation, analgesics, antibiotics, thromboprophylaxis, wound healing, nutritional vulnerability, and organ stress. Elias and colleagues provided early retrospective evidence that surgery in patients receiving ICIs appeared feasible and did not necessarily require stopping ICIs in all contexts, but they also emphasized the need for further confirmation and careful clinical judgment [8].

Perioperative care must therefore become deliberately multidisciplinary. The decision to initiate neoadjuvant or perioperative ICI should include not only medical oncologists and surgeons but also anaesthesiologists, pulmonologists, cardiologists, endocrinologists, radiologists, pathologists, and specialized oncology nurses when clinically indicated. Baseline assessment should identify autoimmune history, pulmonary reserve, endocrine abnormalities, cardiovascular vulnerability, liver function, renal function, infection risk, and corticosteroid exposure. During treatment, clinicians must monitor not only response but also silent toxicities that can become visible only at the moment of surgical stress. After surgery, postoperative complications must be interpreted through an immunological lens, particularly in patients who have recently received PD-1, PD-L1, or CTLA-4 pathway blockade [2,5,6,8,11].

Biomarker development is equally central. Pathological complete response and major pathological response are attractive early endpoints, particularly in neoadjuvant trials, but they do not fully capture the complexity of immune memory, tumor microenvironment remodeling, minimal residual disease and late relapse. The translational work from the SAKK 16/14 cohort showed that tumor immune dynamics can provide clinically relevant insight beyond conventional trial endpoints [3]. In the NADIM trial translational analysis, maintenance of peripheral memory B-cell populations was associated with long-term survival after perioperative chemoimmunotherapy in NSCLC, suggesting that systemic immune signatures may help identify patients most likely to benefit durably [14]. Such findings are particularly important for perioperative care because overtreatment may expose cured patients to unnecessary toxicity, while undertreatment may leave micrometastatic disease unchecked.

Despite rapid progress, several questions remain unresolved. Most pivotal trials were designed to assess pathological response, event-free survival, overall survival, and general surgical feasibility rather than anesthetic techniques, organ-specific perioperative risk, or standardized postoperative surveillance. No universally accepted interval between the final ICI dose and surgery has been established, and practical recommendations are often extrapolated from oncology trials, retrospective cohorts, narrative reviews, case reports, and expert opinion. Furthermore, marked differences in tumor biology, chemotherapy backbone, surgical magnitude, and postoperative complication profiles limit uniform extrapolation across cancer types. A clinically oriented synthesis is therefore needed to distinguish established oncological evidence from indirect perioperative considerations and unresolved research questions.

This scoping review aims to synthesize the current evidence on perioperative care for cancer patients treated with ICIs, with an emphasis on clinical considerations that inform the safe translation of this care into surgical oncology practice. Specifically, it examines surgical timing and feasibility, organ-specific irAEs, postoperative safety, anesthetic considerations, oncological outcomes, and the heterogeneity of these findings across tumor types and surgical procedures. A further objective is to identify which perioperative considerations are supported by direct clinical evidence and which rely primarily on indirect evidence or expert interpretation. By defining these limitations and knowledge gaps, the review seeks to support multidisciplinary decision-making and establish priorities for prospective perioperative and anesthesia-specific research [1,2,3,4,5,6,8,10,11,12,13,14].

## 2. Materials and Methods

### 2.1. Review Design and Reporting Standard

This scoping review was conducted to map the available clinical evidence concerning perioperative care of adult patients with cancer exposed to immune checkpoint inhibitors (ICIs). The review was reported in accordance with the Preferred Reporting Items for Systematic Reviews and Meta-Analyses (PRISMA) 2020 statement and, because of its scoping design, the PRISMA extension for Scoping Reviews (PRISMA-ScR). The protocol was prospectively registered in PROSPERO (CRD420261451278) [15,16].

### 2.2. Eligibility Criteria

Studies were eligible when they: (1) involved adults (≥18 years) with a solid malignancy; (2) evaluated neoadjuvant, adjuvant, or fully perioperative treatment containing an ICI (PD-1, PD-L1, CTLA-4, or related checkpoint blockade); and (3) reported at least one outcome relevant to surgical timing or feasibility, anesthetic or perioperative management, immune-related adverse events (irAEs), postoperative morbidity or mortality, pathological response, or oncological outcome. Randomized and non-randomized trials, prospective and retrospective cohorts, case series, case reports, mechanistic clinical studies, systematic reviews, meta-analyses, and clinically relevant guidance documents were considered because the purpose was evidence mapping. Publications were restricted to the English language and the period from January 2016 through July 2026. Pediatric studies, studies without perioperative or surgical relevance, non-oncology studies, conference items without sufficient clinical data, and duplicate publications were excluded.

For the purposes of this review, the term “perioperative” was used broadly to include ICI treatment before surgery, the intraoperative and postoperative periods, and adjuvant therapy when it directly affected surgical timing, perioperative toxicity, postoperative monitoring, or recovery. This broader definition was chosen to reflect the different treatment schedules reported across the included studies and should not be interpreted as referring exclusively to the conventional immediate perioperative period.

### 2.3. Information Sources and Search Strategy

PubMed/MEDLINE and Web of Science were searched for eligible publications. The search combined controlled vocabulary, where available, and free-text terms describing ICIs, perioperative care, surgery, anesthesia, and cancer. The core concept was as follows: (“immune checkpoint inhibitor” OR immunotherapy OR PD-1 OR PD-L1 OR CTLA-4 OR nivolumab OR pembrolizumab OR atezolizumab OR durvalumab OR avelumab OR ipilimumab) AND (perioperative OR preoperative OR postoperative OR neoadjuvant OR adjuvant OR surgery OR surgical OR anesthesia OR anaesthesia) AND (cancer OR neoplasm OR malignancy). Syntax was adapted to each database. Reference lists of included articles and relevant guidance documents were also searched manually. The final search was completed in July 2026. The complete database-specific strategies are provided in Supplementary Table S1.

### 2.4. Selection Process

**Figure 1:**
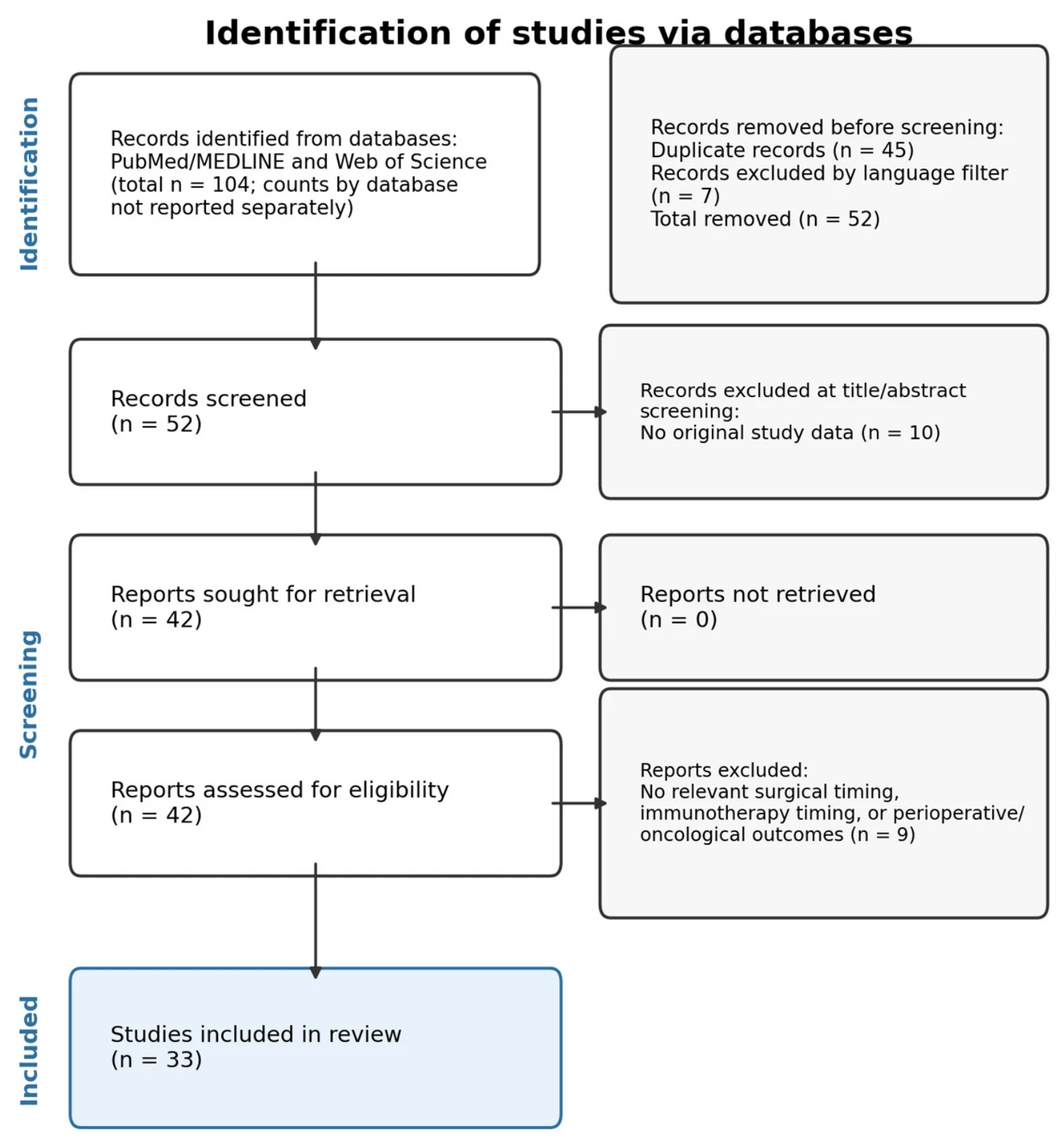
PRISMA 2020 flow diagram of study identification and selection.

All retrieved records were collated, and duplicates were removed before screening. Two reviewers (I.R.C. and L.V.) independently screened the titles and abstracts against the predefined eligibility criteria. Reports considered potentially relevant by either reviewer underwent full-text assessment. The same two reviewers independently assessed the full texts and documented the principal reasons for exclusion. Disagreements at either stage were resolved through discussion and consensus. No automated screening or machine learning prioritization tool was used. The selection process and reasons for full-text exclusion are presented in the PRISMA flow diagram (Figure 1).

### 2.5. Data-Charting Process and Data Items

A standardized data-charting form was developed according to the objectives of the review. The extracted variables included author and publication year, country, study design, tumor type and stage, sample size, ICI agent and therapeutic setting, concomitant systemic therapy, surgical procedure, timing of surgery relative to ICI administration, surgical feasibility, anesthetic and perioperative considerations, irAEs, postoperative outcomes, pathological response, survival outcomes, and the authors’ principal conclusions. One reviewer performed the initial data charting, and the second reviewer checked all entries against the corresponding source publications. Disagreements or uncertainties were resolved by discussion and consensus. The charting framework was refined during the review when additional clinically relevant perioperative variables were identified. Authors of primary reports were not contacted for additional data, and missing information was recorded as not reported.

### 2.6. Critical Appraisal

Consistent with the evidence-mapping objective of a scoping review and the marked heterogeneity of eligible evidence, no formal risk-of-bias assessment was used to exclude studies or generate a pooled certainty estimate. Study design, sample size, directness to perioperative care, and major limitations were nevertheless considered during interpretation, and recommendations were calibrated to the strength and directness of the available evidence. Consequently, the manuscript does not assign formal levels of evidence or grades of recommendation. In the practical recommendations table, the supporting literature is described according to study design and directness to perioperative care. These descriptors are intended to indicate the nature of the available evidence and should not be interpreted as GRADE, Oxford Levels of Evidence, or another validated certainty-of-evidence classification.

### 2.7. Synthesis of Results

The charted evidence was synthesized descriptively and thematically. Studies were first grouped according to cancer type, treatment setting, and study design. Their findings were subsequently mapped across prespecified perioperative domains: timing and feasibility of surgery, anesthetic and immunomodulatory considerations, organ-specific irAEs, postoperative safety outcomes, oncological outcomes, and practical perioperative considerations. Areas of consistency, heterogeneity, and evidence scarcity were identified through structured comparison of the charted data. Because of substantial clinical and methodological heterogeneity in tumor types, therapeutic regimens, study designs, and outcome definitions, no quantitative meta-analysis was performed.

## 3. Results

### 3.1. Study Selection

The database searches identified 104 records. After removal of 45 duplicates and 7 non-English-language records, 52 records underwent title and abstract screening. Ten records without original study data were excluded, leaving 42 reports for full-text assessment; all 42 were retrieved. Nine reports were excluded because they did not provide relevant information on surgical timing, immunotherapy timing, perioperative management, or oncological outcomes. Thirty-three studies were included in the final scoping synthesis (Figure 1).

Unlike previous reviews focusing primarily on oncologic outcomes, the present review systematically synthesizes perioperative variables—including surgical feasibility, anesthetic management, postoperative morbidity, immune-related adverse events, and practical perioperative recommendations—to provide a multidisciplinary perspective for oncologists, surgeons, anesthesiologists, and intensivists.

### 3.2. Study Characteristics

The literature reviewed shows how quickly perioperative immune checkpoint inhibition has progressed, moving from early proof-of-concept studies to recent randomized Phase III clinical trials. The evidence includes a mix of randomized Phase II–III trials, prospective studies, observational cohorts, retrospective studies using propensity score matching, biomarker research, and real-world analyses, along with various reviews and meta-analyses that provide both clinical and mechanistic insights (Supplementary Materials Tables S1 and S2).

The strongest clinical evidence originated from studies in resectable non-small-cell lung cancer (NSCLC). Early feasibility studies, including LCMC3, SAKK 16/14, NADIM, and NEOpredict-Lung [1,2,3,4,14], established the safety and biological activity of neoadjuvant immune checkpoint inhibition, paving the way for pivotal randomized trials such as CheckMate 816, CheckMate 77T, KEYNOTE-671, AEGEAN, RATIONALE-315, and NeoTORCH [1,5,6], which demonstrated significant improvements in pathological response and event-free survival while maintaining acceptable perioperative safety profiles. More recent translational investigations, including the biomarker analyses by Schmid et al. (SAKK 16/14) [3] and Sierra Rodero et al. (NADIM) [13], further expanded the understanding of the immune mechanisms associated with durable responses and long-term survival.

Recent evidence has broadened our understanding of multiple gastrointestinal cancers. In studies focused on gastric and gastroesophageal junction adenocarcinoma, notable trials such as PHERFLOT [8] and DRAGON IV/CAP05 [9] have shown that combining treatments, including pembrolizumab and trastuzumab, with FLOT can lead to improved pathological responses while maintaining the feasibility of surgery. Additional insights came from the Phase III study involving camrelizumab and rivoceranib, as well as research on how immune modulation and anesthetic methods, including opioid-free anesthesia, can affect outcomes.

Studies have explored the use of perioperative immunotherapy in esophageal squamous cell carcinoma, showing promising results with high rates of complete tumor removal and positive responses after neoadjuvant chemoimmunotherapy. These findings also provided valuable insights for surgical planning. This trend is also seen in muscle-invasive bladder cancer, as well as in breast cancer (KEYNOTE-522), ovarian cancer [10,11,12,13], and others. Notably, a real-world study by Wang et al. reported impressive response rates and encouraging long-term survival among patients receiving immune checkpoint therapy for these cancers [6].

Recent studies have highlighted the importance of perioperative management alongside advancements in cancer treatment. Research by Elias et al. [7], Tong et al. [14], and others has shown that surgeries after neoadjuvant immunotherapy are feasible with low complication rates. Additionally, work by Wang et al. [17], Liu et al. [18], and Rose et al. [10] has focused on how anesthetic techniques and immune modulation can affect recovery and long-term cancer outcomes.

Finally, the growing body of clinical evidence has been synthesized by several high-quality reviews, including those by Lazzari et al. [1], Sandbank et al. [19], Tang et al. [20], Ackerman et al. [21], Ciner et al. [22], and Cao et al. [23], which collectively highlight the multidisciplinary challenges of perioperative immunotherapy, the importance of immune-related adverse events, and the need for standardized perioperative pathways. Together, these publications illustrate the transition from isolated feasibility studies to an increasingly mature evidence base supporting the integration of immune checkpoint inhibitors into curative-intent multimodal cancer treatment, while emphasizing that important questions regarding optimal perioperative management, anesthetic strategies, and long-term safety remain unresolved.

### 3.3. Surgical Timing and Feasibility Following Neoadjuvant Immunotherapy

The timing of surgery following immune checkpoint inhibitor (ICI) therapy varied according to the treatment strategy and tumor type, reflecting the absence of a universally accepted perioperative schedule. In most neoadjuvant and perioperative protocols, surgery was performed after completion of two to four cycles of immunotherapy, administered either alone or in combination with platinum-based chemotherapy, whereas postoperative maintenance immunotherapy was continued for several months in studies that adopted a true perioperative approach. Despite variations in study design, surgery was generally scheduled once patients had recovered from induction therapy and no clinically significant immune-related adverse events (irAEs) were present.

The pivotal randomized NSCLC trials—including CheckMate 816, CheckMate 77T, KEYNOTE-671, RATIONALE-315, NeoTORCH, NADIM, and NADIM II [1,5,6,14]—consistently demonstrated that neoadjuvant chemoimmunotherapy did not compromise the feasibility of subsequent surgical resection. High rates of protocol completion and R0 resection were achieved despite the addition of immunotherapy, supporting the integration of ICIs into standard multimodal treatment pathways. Earlier feasibility studies, including LCMC3, SAKK 16/14, and NEOpredict-Lung, similarly confirmed that surgery could be safely performed following neoadjuvant checkpoint inhibition and provided the foundation for subsequent randomized investigations [1,2,4].

Real-world thoracic surgical studies have further validated these results. Guo et al. (2024) [24] administered two to four cycles of a neoadjuvant PD-1 inhibitor alongside platinum-based chemotherapy prior to lobectomy, while Pan et al. (2024) [25] noted a median interval of 35 days between finishing neoadjuvant immunochemotherapy and surgery for patients undergoing either video-assisted thoracoscopic surgery (VATS) or thoracotomy. Similarly, Tong et al. (2022) [14], Zhang et al. (2025) [26], Chen et al. (2025) [27], and Tan et al. (2026) [28] found that neoadjuvant immunotherapy rarely led to treatment discontinuation or significant delays in surgery, although there were instances where inflammatory changes and fibrosis increased the complexity of the procedures.

Recent studies have shown that similar treatment plans are being used for various gastrointestinal cancers. For example, in the MONEO trial, patients underwent four cycles of neoadjuvant avelumab combined with FLOT chemotherapy before their curative gastrectomy. Afterward, they received an additional four cycles of treatment along with maintenance avelumab for up to a year (Alsina et al., 2025) [11]. This kind of perioperative strategy was also seen in the PHERFLOT study [29] and the DRAGON IV/CAP05 trial [30]. Additionally, multicenter retrospective studies by Sun et al. and Cui et al. revealed that incorporating neoadjuvant PD-1-based therapy into surgical treatments can be done safely, without increasing surgical risks. This approach is not limited to gastrointestinal cancers; similar methods have been explored in cases of hepatocellular carcinoma, muscle-invasive bladder cancer (like in the PURE-01, ABACUS, and VESTIGE trials) [1,31], dMMR/MSI-H colorectal cancer, and even ovarian cancer. These findings highlight how versatile perioperative immunotherapy can be across various solid tumors.

In contrast to neoadjuvant protocols, some studies investigated exclusively postoperative immunotherapy. The VESTIGE trial initiated nivolumab plus ipilimumab within three months after surgery in patients with high-risk gastroesophageal adenocarcinoma and continued treatment for up to one year (Lordick et al., 2025) [31]. Likewise, Moore et al. (2021) [12] employed a flexible strategy in ovarian cancer, administering atezolizumab either after primary cytoreductive surgery or after neoadjuvant chemotherapy, followed by adjuvant and maintenance treatment.

From the perspective of perioperative medicine, the definition of ICI exposure also varied substantially. Tang et al. (2024) [20] proposed the broadest perioperative definition, considering any FDA-approved checkpoint inhibitor administered within 180 days before surgery as clinically relevant. This approach recognizes that immune-related adverse events may develop weeks or even months after treatment discontinuation and therefore remain important throughout the perioperative period [20].

Overall, the available evidence consistently indicates that the incorporation of immune checkpoint inhibitors into neoadjuvant treatment does not preclude timely surgery or reduce surgical feasibility. Nevertheless, no standardized interval between the final ICI dose and surgery has been established, and perioperative decision-making continues to rely on multidisciplinary assessment of tumor characteristics, recovery from induction therapy, and the presence or resolution of immune-related toxicities.

### 3.4. Immune-Related Adverse Events Relevant to Perioperative Management

Immune checkpoint inhibitors can lead to a range of immune-related side effects that impact nearly every organ system, making perioperative management crucial. The severity of these side effects often depends on the treatment approach, with combination therapies usually resulting in more serious issues than single-agent treatments. While most side effects can be managed with supportive care and immunosuppressive therapy, some severe reactions may delay treatment or necessitate careful decision-making and longer-term management.

#### 3.4.1. Endocrine Toxicities

Endocrine-related immune adverse events (irAEs) are commonly reported toxicities in various studies. Conditions like hypothyroidism, hyperthyroidism, adrenal insufficiency, hypophysitis, and thyroiditis have been noted in trials such as VESTIGE, MONEO, and KEYNOTE-522, as well as in the ovarian cancer research by Moore et al. Although most of these events are mild (grade 1–2), they are crucial to recognize in the perioperative context, as they can cause vague symptoms like fatigue, low blood pressure, low sodium levels, or confusion. Experts, including Ackerman et al. and Sandbank et al., stress the importance of preoperative endocrine assessments and early detection of adrenal insufficiency to prevent serious complications during surgery.

#### 3.4.2. Pulmonary Toxicities

Pulmonary toxicity remains one of the most clinically significant complications for thoracic surgeons and anesthesiologists. Immune-mediated pneumonitis was reported across multiple NSCLC trials—including CheckMate 816, CheckMate 77T, KEYNOTE-671, AEGEAN, RATIONALE-315, and NeoTORCH—although its incidence was generally low. Nevertheless, reviews by Ackerman et al., Sandbank et al., and Tang et al. emphasize that even low-grade pneumonitis may impair pulmonary reserve and increase the risk of postoperative respiratory complications, particularly following major lung resection.

#### 3.4.3. Gastrointestinal and Hepatic Toxicities

Immune-mediated colitis, diarrhea, hepatitis, and transaminase elevation were among the most frequently reported non-endocrine toxicities. In the MONEO trial, grade ≥ 3 treatment-related adverse events occurred in 80% of patients, although severe avelumab-related toxicities were limited to 25%; common adverse events included diarrhea, nausea, vomiting, fatigue, neutropenia, and elevated liver enzymes, without treatment-related mortality. Similarly, VESTIGE reported gastrointestinal and hepatic irAEs, including colitis and transaminase elevation, as well as endocrine manifestations. Comparable hepatic toxicities were also described in PHERFLOT, DRAGON IV/CAP05, and several gastric cancer cohorts, although most were reversible following corticosteroid therapy and treatment interruption.

#### 3.4.4. Dermatologic and Infusion-Related Toxicities

Cutaneous reactions, including rash and pruritus, together with infusion-related reactions, were among the most common low-grade irAEs. These events were consistently reported in the MONEO, VESTIGE, Moore et al., KEYNOTE-522, and several NSCLC trials, and rarely required permanent treatment discontinuation, but frequently necessitated symptomatic management.

#### 3.4.5. Neurological and Cardiovascular Toxicities

Although uncommon, neurological and cardiovascular irAEs remain the most feared complications because of their potentially life-threatening nature. The ovarian cancer trial by Moore et al. reported a fatal case of immune-mediated myasthenia gravis, illustrating the catastrophic consequences of rare neuromuscular toxicity. Likewise, Ackerman et al., Sandbank et al., and Tang et al. identify myocarditis, conduction abnormalities, encephalitis, peripheral neuropathies, and myasthenic syndromes as uncommon but critical complications that require prompt multidisciplinary recognition and management.

### 3.5. Perioperative Implications

The clinical signs of immune-related adverse events can often mimic common postoperative complications, making diagnosis tricky. For instance, Ciner et al. recounted the case of a patient who had a successful lung surgery after treatment with nivolumab but later developed fever, low blood pressure, respiratory issues, kidney injury, and shock. Initially thought to be postoperative sepsis, it turned out to be cytokine release syndrome and immune-related toxicity, which responded well to corticosteroids. Tang et al. defined preoperative ICI exposure as treatment received within 180 days before surgery. This interval was an epidemiological exposure window used in their retrospective analysis and should not be interpreted as a recommendation for intensive surveillance of every patient throughout the entire six-month period. In clinical practice, a history of ICI exposure during this interval should prompt documentation of the agent, last dose, previous irAEs, and current symptoms. A practical approach is therefore to document the ICI agent, date of the last dose, combination therapy, previous irAEs, immunosuppressive treatment, and unresolved organ dysfunction. The timing of surgery and intensity of postoperative monitoring should be individualized based on clinical recovery, active or prior toxicity, concomitant therapy, surgical extent, and physiological risk, rather than determined solely by a fixed number of days after the last ICI dose. Additional investigations and higher-acuity postoperative monitoring should be individualized based on active or prior toxicity, comorbidities, surgical extent, and clinical findings. These examples highlight the importance of careful postoperative monitoring and teamwork among surgeons, anesthesiologists, intensivists, and oncologists to differentiate irAEs from typical postoperative issues. The principal organ-specific immune-related adverse events and their implications for anesthetic management are summarized in Table 1.

### 3.6. Anesthetic Implications of Perioperative Immune Checkpoint Inhibition

Although relatively few studies were specifically designed to evaluate anesthetic management, the available literature provides several clinically relevant considerations for perioperative physicians. Collectively, the evidence suggests that immune checkpoint inhibitors should not be regarded as a contraindication to surgery or anesthesia. Instead, successful perioperative management depends on meticulous preoperative assessment, anticipation of immune-related toxicities, individualized intraoperative planning, and multidisciplinary postoperative surveillance (Ackerman et al., 2022; Sandbank et al., 2023; Tang et al., 2024) [19,20,21].

#### 3.6.1. Preoperative Assessment

A recurring finding across both clinical studies and narrative reviews is the importance of identifying prior ICI exposure before surgery. In addition to documenting the checkpoint inhibitor, treatment duration, and interval from the last dose, clinicians should actively screen for previous or ongoing immune-related adverse events. Current evidence supports routine evaluation of pulmonary, endocrine, cardiac, hepatic, and nutritional status before major oncologic surgery, particularly in patients treated with combination chemoimmunotherapy or dual checkpoint blockade (KEYNOTE-671; RATIONALE-315; DRAGON IV/CAP05; PHERFLOT; VESTIGE; Tang et al.; Ackerman et al.; Sandbank et al.; Björkström et al.) [5,18,19,20,21,30,31,32,33].

Particular attention should be directed toward endocrine dysfunction, including hypothyroidism, hypophysitis, and adrenal insufficiency, as these conditions may remain clinically silent until the physiological stress of surgery precipitates refractory hypotension or delayed recovery. Likewise, preoperative pulmonary assessment is especially important before thoracic procedures because previous immune-mediated pneumonitis or reduced diffusion capacity may increase postoperative respiratory risk (Zhang et al.; Tan et al.; KEYNOTE-671; RATIONALE-315) [5,6,26,28].

#### 3.6.2. Intraoperative Considerations

While standard anesthetic methods work well for most patients receiving immune checkpoint inhibitors, there are important things to take into consideration. Research by Tang et al. points out that these patients may experience issues like prolonged reliance on vasopressors, the need for extra oxygen, and potential heart injury during surgery. This suggests that those who have received ICIs might be more vulnerable when it comes to heart and lung health in the perioperative period [20].

Several studies in thoracic surgery, including work by Pan and colleagues, Tong’s team, Zhang, and Tan, have shown that neoadjuvant immunotherapy does not rule out the option of minimally invasive surgery [17,26,26,28]. However, it is worth noting that this approach can lead to increased tissue inflammation and fibrosis. This occasionally complicates procedures, like hilar dissection, and raises the risk of needing to switch to a more invasive thoracotomy. Given these challenges, it is clear that having a specialized thoracic anesthetic management plan is crucial for these patients. This includes carefully managing one-lung ventilation, implementing lung-protective ventilation strategies, and customizing fluid therapy to meet individual needs.

Recent research is showing that how we manage pain during surgery might play a key role in recovery afterward. Studies from Liu and colleagues, Wang and team, and Hu and others have found that using a combination of different pain relief methods, especially those that reduce the use of opioids, could help lessen the suppression of the immune system that sometimes happens during surgery. These approaches provide good pain control but, at this point, we still do not have solid evidence that they lead to better long-term outcomes for cancer patients [18,19,34].

#### 3.6.3. Postoperative Management

The postoperative period can be challenging for patients who have recently received immune checkpoint inhibitors. As highlighted by Ciner et al., immune-related side effects can mimic serious conditions like sepsis, respiratory failure, or organ dysfunction [22]. For instance, a patient exhibiting recurrent fever, low blood pressure, and kidney issues was initially suspected of having an infection, but it turned out to be cytokine release syndrome, which responded well to corticosteroids.

Tang et al. also noted that anyone who has been treated with an FDA-approved checkpoint inhibitor in the last 180 days should be monitored for delayed immune-related complications. Therefore, if a patient experiences unexplained low blood pressure, breathing difficulties, heart irregularities, or kidney and hormonal issues after surgery, it is essential to consider immune-related toxicity alongside usual surgical risks [20].

Overall, this situation calls for a collaborative approach involving surgeons, anesthesiologists, intensivists, oncologists, and other specialists to ensure rapid diagnosis and appropriate immunosuppressive treatment when necessary. This teamwork helps maintain patient safety and supports the continuation of cancer treatment.

### 3.7. Oncologic Outcomes

Research shows that using immune checkpoint inhibitors (ICIs) alongside standard treatment can enhance pathological response rates across various solid tumors without complicating surgery. However, the level of benefit depends on factors like tumor type, treatment approach, disease stage, and biomarker status.

#### 3.7.1. Pathological Response

Pathological complete response (pCR) and major pathological response (MPR) are key indicators commonly used to assess treatment outcomes. In resectable non-small-cell lung cancer (NSCLC), several major trials, such as CheckMate 816 [1] and KEYNOTE-671 [6], have shown that patients receiving neoadjuvant chemoimmunotherapy have significantly higher response rates than those receiving chemotherapy alone. Real-world studies by researchers such as Pan and Zhang support these findings, indicating that the results hold true outside clinical trials.

Similar patterns have emerged in gastrointestinal cancers as well. For instance, the MONEO trial found that combining avelumab with FLOT resulted in a pCR rate of 21.1% and an MPR rate of 28.9%, particularly in tumors with higher PD-L1 levels [12]. Other studies, like the PHERFLOT trial, have echoed these positive results, confirming that perioperative immunotherapy can lead to promising pathological response [30]. In cases of pulmonary sarcomatoid carcinoma, one study reported that among four patients treated, one achieved a complete response and all were disease-free at the latest follow-up.

#### 3.7.2. Survival Outcomes

Recent studies have shown that while early responses to treatment are important, long-term survival is the ultimate goal for assessing success. Data from trials like CheckMate 816 and CheckMate 77T indicate that using immune checkpoint inhibitors in the perioperative setting for resectable non-small cell lung cancer (NSCLC) leads to better survival rates. Notably, the NADIM trial highlighted strong long-term outcomes with neoadjuvant chemoimmunotherapy. Researchers have found that favorable immune microenvironments, characterized by increased immune cell activity, are linked to these durable survival rates.

In gastrointestinal cancers, the MONEO trial reported impressive three-year survival rates of 66% for progression-free survival and 69% for overall survival [12]. Other studies also show promising results in controlling disease, though data continues to evolve. In muscle-invasive bladder cancer, trials like PURE-01 and ABACUS have shown encouraging responses and recurrence rates, suggesting that immunotherapy could play a vital role in treating these conditions [11].

#### 3.7.3. Heterogeneity of Treatment Benefit

Despite these encouraging findings, not all studies demonstrated a survival advantage. In the large randomized ovarian cancer trial by Moore et al., the addition of atezolizumab to standard chemotherapy failed to improve progression-free or overall survival in the overall study population, with benefit limited to an exploratory subgroup exhibiting high PD-L1 expression. Similarly, the VESTIGE trial demonstrated inferior disease-free and overall survival with adjuvant nivolumab plus ipilimumab compared with continuation of chemotherapy in patients with high-risk resected gastroesophageal adenocarcinoma, leading the investigators to discourage routine substitution of postoperative chemotherapy with dual checkpoint blockade in unselected patients.

Collectively, these findings indicate that perioperative immunotherapy has established substantial oncologic benefit in several malignancies—particularly resectable NSCLC—but that therapeutic efficacy remains heterogeneous across tumor types and treatment settings. The contrasting results observed in studies such as VESTIGE and Moore et al. underscore the importance of appropriate patient selection and reinforce the growing role of predictive biomarkers, including PD-L1 expression, molecular subtype, pathological response, and emerging immune microenvironment signatures, in optimizing perioperative treatment strategies [12,32].

### 3.8. Practical Recommendations

Although prospective anesthesia-specific evidence remains limited, the synthesized literature supports several actionable, multidisciplinary recommendations for the perioperative management of cancer patients treated with ICIs. These are intended as pragmatic guidance rather than formal practice standards, given the current maturity of the evidence base (Table 2).

**Figure 2:**
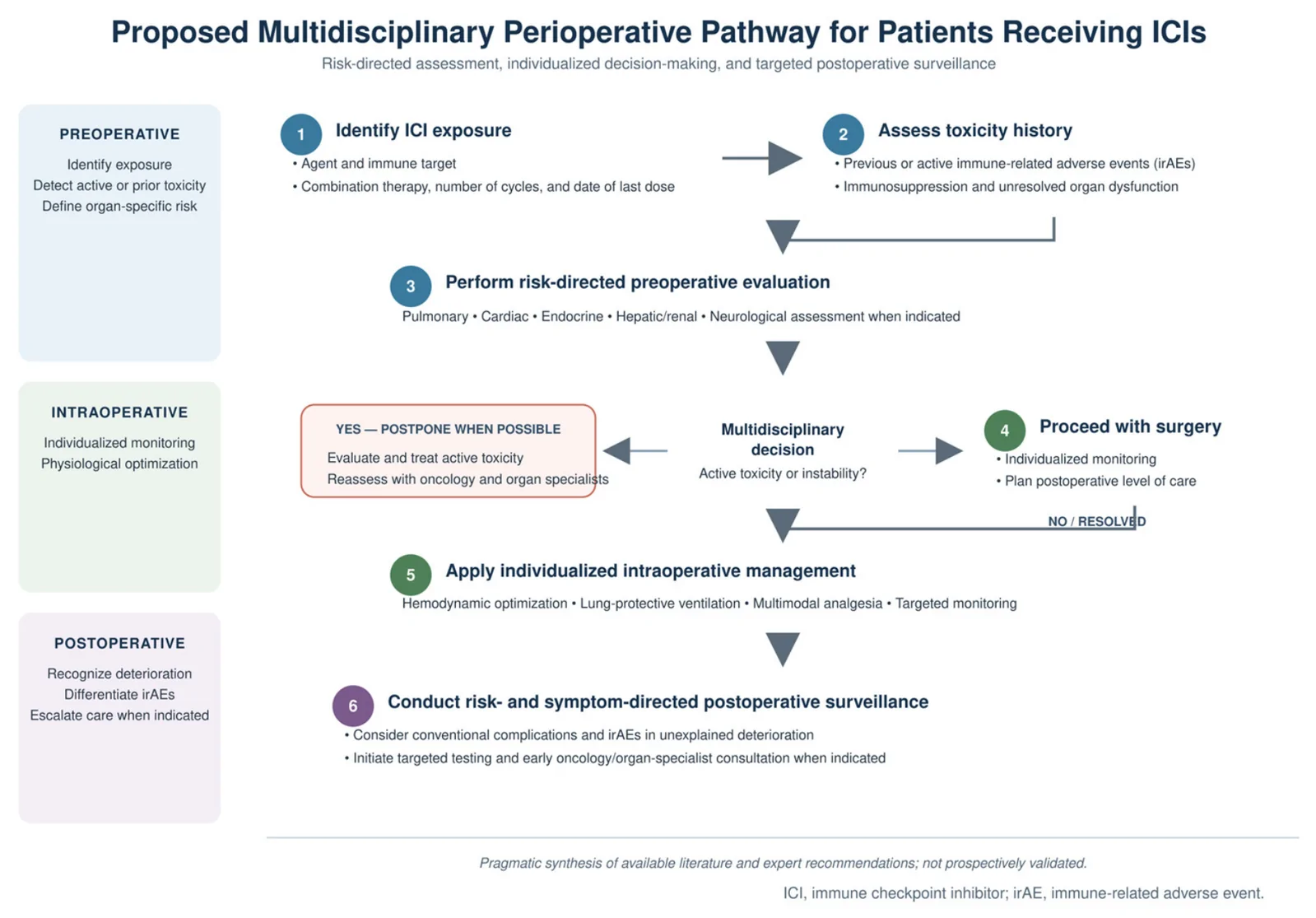
Proposed multidisciplinary perioperative pathway for patients receiving immune checkpoint inhibitors. The pathway represents a pragmatic synthesis of the available literature and expert recommendations and has not been prospectively validated. (The image was imagined by the authors and created with AI).

The pathway summarizes ICI exposure assessment, toxicity screening, risk-directed preoperative evaluation, multidisciplinary decision-making, intraoperative management, and postoperative surveillance. It represents a pragmatic synthesis of the available literature and expert recommendations and has not been prospectively validated. (ICI, immune checkpoint inhibitor; irAE, immune-related adverse event; Figure 2).

## 4. Discussion

Immune checkpoint inhibitors have changed the game for treating solid cancers. What started as a treatment for advanced cases is now being used preoperatively, showing promise in improving response rates and long-term outcomes without compromising surgical safety. Landmark clinical trials, including CheckMate 816, CheckMate 77T, KEYNOTE-671, AEGEAN, RATIONALE-315, NeoTORCH, NADIM, and NADIM II, have established perioperative chemoimmunotherapy as a new therapeutic paradigm for resectable NSCLC [1,5,6,14], while studies such as PHERFLOT, DRAGON IV/CAP05, MONEO, PURE-01, ABACUS, and KEYNOTE-522 have expanded these concepts to gastrointestinal, urothelial, and breast malignancies [10,12,31]. Collectively, these studies demonstrate that immune checkpoint inhibition has evolved from an experimental strategy into an integral component of multimodal cancer therapy.

One of the most important insights from this review is that we need to rethink our view of perioperative immunotherapy. It is no longer just about the effectiveness of fighting cancer. While we still focus on metrics such as pathological complete response and event-free survival in clinical trials, the reality of managing patients during this time has become much more complex. Today, surgery following neoadjuvant immunotherapy is the norm, not the exception. This shift calls for stronger collaboration among anesthesiologists, surgeons, intensivists, and oncologists, as they need to understand the unique effects of immune checkpoint blockade on patients. Instead of seeing the perioperative period as just a gap between treatments, we should recognize it as a vital part of comprehensive cancer care.

A consistent finding across both randomized trials and real-world surgical cohorts is that prior ICI exposure rarely compromises surgical feasibility. High protocol completion rates, excellent R0 resection rates, and acceptable postoperative morbidity were reported across thoracic, gastric, esophageal, hepatobiliary, and urothelial surgery. Studies by Pan et al., Tong et al., Guo et al., Chen et al., Zhang et al., and Tan et al. confirmed that minimally invasive approaches remain feasible despite occasional increases in hilar fibrosis, tissue edema, and inflammatory adhesions [14,24,25,26,27,28]. Similarly, pivotal randomized NSCLC trials consistently demonstrated that neoadjuvant chemoimmunotherapy neither substantially delays surgery nor increases perioperative mortality. These observations collectively challenge the initial concern that immune activation might impair operability or substantially increase surgical risk. Instead, they suggest that careful patient selection and multidisciplinary planning are considerably more important than arbitrary treatment-free intervals before surgery.

One of the most significant insights from this review is the focus on anesthetic and perioperative considerations. Unlike earlier reviews that primarily examined factors such as cancer treatment effectiveness, pathological responses, or biomarkers, we have highlighted the importance of perioperative medicine, which is often overlooked. Our findings reveal that effective perioperative management goes beyond the standard preoperative check-up; it requires a thorough evaluation for possible organ-specific immune-related adverse events (irAEs).

The potential for complications in various areas—such as the lungs, endocrine system, heart, liver, kidneys, nervous system, and gastrointestinal tract—can significantly affect anesthetic care, even if these issues are not apparent before surgery. Therefore, it is essential to include the patient’s history of immune checkpoint inhibitor (ICI) exposure in routine preoperative assessments, as we do for prior chemotherapy or radiotherapy. This proactive approach could pave the way for safer and more effective surgical experiences [9,13,37,38,39].

Endocrine dysfunction, a type of immune-related toxicity, is particularly noteworthy because it can go unnoticed until surgery reveals issues like adrenal insufficiency or hypophysitis. Research, including reviews by Ackerman et al., Sandbank et al., Tang et al., and Björkström et al., highlights that signs such as persistent low blood pressure, unexplained low sodium levels, extended need for vasopressors, or slow recovery should raise concerns about hidden endocrine issues, instead of attributing them to bleeding, infections, or anesthesia problems. Additionally, while less common, immune-related heart and lung inflammation can be serious, often mimicking typical postoperative complications but carrying higher risks. These findings underscore the importance of a thorough evaluation by a multidisciplinary team when patients with prior immune checkpoint inhibitor treatments are preparing for major cancer surgery [19,20,21,32].

The postoperative period can be quite tricky for diagnosing complications. A notable case by Ciner et al. highlighted how cytokine release syndrome mimicked severe postoperative sepsis, showing up as fever, shock, respiratory failure, and kidney injury [22]. Likewise, Tang et al. noted that significant immune-related adverse events (irAEs) might emerge weeks or even months after the last dose of immunotherapy [20]. They advised that patients who received checkpoint inhibitors in the past 180 days should be closely monitored. These insights underscore the importance of considering immune-related issues when patients worsen after surgery, rather than just attributing it to typical surgical problems [21].

Another important observation emerging from this review concerns the remarkable heterogeneity of oncologic benefit across different malignancies. While randomized trials in resectable NSCLC consistently demonstrated substantial improvements in pathological response and survival, results from other tumor types have been more variable. The MONEO trial demonstrated encouraging pathological responses and favorable three-year survival [12], whereas the VESTIGE trial failed to demonstrate superiority of adjuvant nivolumab–ipilimumab over postoperative chemotherapy [32]. Likewise, the ovarian cancer trial by Moore et al. failed to improve survival despite acceptable safety, with benefit confined to a PD-L1-high subgroup. These contrasting findings illustrate that immune checkpoint inhibition cannot yet be considered universally beneficial across all tumor types and highlight the importance of individualized treatment selection.

The growing importance of biomarkers represents another major theme emerging from the literature. Although PD-L1 expression currently remains the most widely used predictive biomarker in clinical practice, accumulating translational evidence suggests that treatment response depends upon a considerably more complex interaction between tumor biology and host immunity. Biomarker analyses from SAKK 16/14, NADIM, and other translational studies identified tertiary lymphoid structures, CD8^+^ tumor infiltration, T-cell receptor diversity, circulating immune cell populations, cytokine signatures, and dynamic changes in the tumor microenvironment as promising predictors of durable therapeutic benefit. These observations are consistent with broader reviews that describe the rapidly evolving understanding of antitumor immunity and suggest that future treatment algorithms will increasingly integrate molecular biomarkers with clinicopathological variables to personalize perioperative immunotherapy [1,2].

### 4.1. Tumor- and Procedure-Specific Perioperative Heterogeneity

The perioperative implications of ICI exposure cannot be assumed to be uniform across tumor types and surgical procedures. Differences in tumor biology, treatment combinations, organ-specific toxicity, surgical magnitude, and postoperative complication profiles substantially affect clinical interpretation. Therefore, findings from one oncological setting should be extrapolated to another with caution.

In resectable NSCLC, the principal perioperative concerns are pulmonary reserve, prior or subclinical pneumonitis, treatment-related changes in diffusion capacity, and the technical consequences of hilar inflammation or fibrosis. These factors are particularly relevant during lung resection requiring one-lung ventilation, when immune-mediated pulmonary injury must be differentiated from atelectasis, pneumonia, aspiration, pulmonary edema, and pulmonary embolism. The relatively mature evidence base in NSCLC supports surgical feasibility after neoadjuvant chemoimmunotherapy, but it does not eliminate the need for procedure-specific pulmonary assessment and postoperative respiratory surveillance.

Upper gastrointestinal surgery presents a different diagnostic context. After gastrectomy or esophagectomy, fever, abdominal pain, diarrhea, hemodynamic instability, and elevated inflammatory markers may indicate anastomotic leakage, intra-abdominal infection, ischemic complications, chemotherapy-related toxicity, or immune-mediated enterocolitis. Because delayed recognition of a surgical complication may be catastrophic, conventional postoperative complications must be actively excluded first, while immune-mediated toxicity remains part of the differential diagnosis. Nutritional impairment, hepatic toxicity, corticosteroid exposure, and the intensity of combined chemotherapy may further influence recovery in this population.

In breast cancer, muscle-invasive bladder cancer, ovarian cancer, and other solid tumors, perioperative risk is also shaped by the accompanying chemotherapy regimen, surgical extent, reconstructive procedures, thromboembolic risk, renal function, and postoperative treatment plan. However, anesthesia- and surgery-specific evidence in these populations remains substantially less developed than in NSCLC. The available findings should therefore be interpreted within their tumor- and procedure-specific context rather than used to support a single uniform perioperative strategy for all patients receiving ICIs.

Overall, the common principle across tumor types is not a standardized anesthetic technique or a fixed interval before surgery, but structured documentation of ICI exposure, identification of previous or active irAEs, procedure-specific risk assessment, and multidisciplinary planning. The intensity of preoperative investigation and postoperative monitoring should be determined by the affected organ systems, the magnitude of surgery, the concomitant treatment regimen, and the patient’s physiological reserve.

The evolution of immunotherapy raises important questions for anesthetic research. Right now, most recommendations about anesthesia during surgery are based on cancer studies and expert opinions rather than direct research focused on anesthesia. This leaves us with many unanswered questions: Do specific anesthetic techniques affect immune responses after checkpoint blockade? Does using fewer opioids lead to better long-term cancer outcomes? Can personalized hemodynamic management during surgery help reduce immune-related complications afterward? We are also still figuring out the best criteria for when patients should go to intensive care after surgery and how long they should be monitored. Addressing these gaps is crucial for future research.

This review has some notable strengths. Unlike earlier studies that mainly focused on cancer treatment effectiveness, we took a broader look at perioperative factors, including when surgery is performed, its feasibility, anesthetic approaches, immune-related side effects, postoperative complications, and practical recommendations. This multidisciplinary approach aims to improve communication among oncologists, surgeons, anesthesiologists, and intensivists, thereby creating standardized pathways for patients receiving immune checkpoint inhibitors.

### 4.2. Knowledge Gaps and Future Research Priorities

Despite increasing research on immunotherapy (ICI) for cancer, key gaps remain in surgical and anesthesia management.

#### 4.2.1. Timing of Surgery

There is no established optimal timing between the last ICI treatment and surgery. Many clinical trial protocols scheduled surgery approximately four to six weeks after completion of neoadjuvant therapy; however, this interval has not been validated as a universal safety threshold across ICI agents, treatment combinations, tumor types, and surgical procedures. More research across different cancer types through prospective studies is needed to determine whether optimal intervals differ by ICI class, combination therapy, tumor type, and surgical complexity.

#### 4.2.2. Anesthetic Management

Anesthetic management for ICI patients is mostly based on theory rather than clinical evidence. The effects of intravenous versus inhalational anesthesia, regional techniques, opioid exposure, and perioperative corticosteroid administration on immune function and long-term oncological outcomes require dedicated investigation. Therefore, the anesthesia-related considerations presented in this review are primarily hypothesis-generating and should not be interpreted as evidence that a particular anesthetic technique improves immune or oncological outcomes.

#### 4.2.3. Outcome Definitions and Surveillance

Safety outcomes are inconsistently defined. Recent findings show increased vasopressor use and cardiovascular issues, which require further investigation. Standardized definitions are needed for postoperative irAEs, cardiopulmonary instability, vasopressor dependence, respiratory complications, acute kidney injury, intensive care admission, surgical delay, and postoperative mortality. The appropriate duration of postoperative surveillance also remains uncertain because immune-mediated complications may occur after hospital discharge.

#### 4.2.4. Underrepresented Populations

Specific groups, such as the elderly, need more focus. While they benefit from ICI, there are no clear guidelines for their care leading up to and after surgery. More research is needed to ensure safe and effective perioperative management for all patients on ICIs [13,32].

Future research should focus on prospective, multidisciplinary studies that utilize standardized reporting for perioperative immunotherapy exposure, monitoring of immune-related adverse events (irAEs), anesthetic techniques, and both short-term surgical outcomes and long-term oncologic results. There is a critical need to develop and validate standardized perioperative care pathways for patients receiving immune checkpoint inhibitors (ICIs).

### 4.3. Limitations

This review comes with a few important limitations to keep in mind. As a scoping review, its main goal was to explore and outline the wide range of available evidence rather than to conduct a detailed statistical analysis. Because the studies varied greatly in design, tumor types, immunotherapy approaches, and outcome definitions, we were not able to perform a formal meta-analysis. The studies included were quite diverse, ranging from individual case reports to large randomized trials. Much of the information on perioperative care and anesthesia was obtained indirectly from studies focused on oncologic effectiveness rather than on perioperative outcomes. Unfortunately, there were not many anesthesia-specific studies available, so we drew some conclusions based on narrative reviews and theoretical reasoning rather than solid, controlled evidence.

We also need to acknowledge potential biases in the publication and language, as our search was limited to English-language articles. Lastly, this field is changing rapidly, which means that some newer trials and treatments—like combination therapies and the latest checkpoint inhibitors—might not be fully captured in this review.

## 5. Conclusions

Perioperative and neoadjuvant immunotherapy has become an established component of multimodal cancer care, and the available evidence indicates that surgery following ICI exposure is generally feasible and safe, without a consistent increase in postoperative mortality or overall surgical complications. Nevertheless, important uncertainties persist. The optimal timing of surgery relative to ICI administration remains undefined; irAEs may arise or persist well into the postoperative period and can closely mimic conventional surgical complications, and anesthesia-specific evidence—particularly regarding opioid interactions, regional techniques, and hemodynamic stability—remains immature. Signals of prolonged vasopressor dependence, cardiovascular instability, and increased perioperative opioid requirements highlight the need for heightened perioperative vigilance. Optimal care depends on structured preoperative screening for irAEs, individualized timing decisions, careful corticosteroid management, vigilant postoperative surveillance, and close multidisciplinary collaboration. Available subgroup evidence in resectable NSCLC suggests that patients aged ≥65 years may derive oncological benefit from perioperative immunotherapy; however, age alone should not determine eligibility, and treatment decisions should incorporate frailty, comorbidities, functional status, organ reserve, and the limited age-specific perioperative safety evidence. Ultimately, prospective, multidisciplinary studies with standardized reporting of perioperative immunotherapy exposure and outcomes are needed to translate this rapidly expanding oncologic paradigm into safe, evidence-based perioperative practice.

## Figures and Tables

**Table 1:** Immune-related adverse events relevant to anesthetic management. Abbreviations: ACTH, adrenocorticotropic hormone; BNP, B-type natriuretic peptide; DLCO, diffusing capacity of the lung for carbon monoxide; ECG, electrocardiography; ICU, intensive care unit; irAE, immune-related adverse event; PFT, pulmonary function test.

irAE	Clinical Relevance for Anesthesia	Suggested Perioperative Assessment	Intra/Postoperative Concern
Pneumonitis	Hypoxemia, reduced pulmonary reserve	Symptoms, CT/CXR, PFTs/DLCO	Difficult ventilation, postoperative respiratory failure
Myocarditis	Arrhythmias, cardiogenic shock	ECG, troponin, BNP, echocardiography	Hemodynamic collapse
Adrenal insufficiency/Hypophysitis	Refractory hypotension	Morning cortisol, ACTH	Stress-dose steroids
Thyroid dysfunction	Bradycardia/tachycardia	TSH, free T4	Delayed recovery
Hepatitis	Drug metabolism	AST/ALT, bilirubin	Avoid hepatotoxic drugs
Colitis	Fluid loss, electrolyte imbalance	GI symptoms	Hypovolemia, sepsis mimic
Nephritis	AKI	Creatinine, eGFR	Drug dose adjustment
Neurologic irAEs	Myasthenia, encephalitis	Neurologic examination	Prolonged neuromuscular blockade
Type 1 diabetes	Hyperglycemia/DKA	Blood glucose	ICU if DKA
Dermatologic toxicity	Usually mild	Skin examination	Infection/wound care

**Table 2:** Practical Perioperative Recommendations for Patients Receiving Immune Checkpoint Inhibitors Extracted from the Literature. Abbreviations: CRS, cytokine release syndrome; DLCO, diffusing capacity of the lung for carbon monoxide; ECG, electrocardiography; ICI, immune checkpoint inhibitor; ICU, intensive care unit; irAE, immune-related adverse event; PD-1, programmed cell death protein 1; VATS, video-assisted thoracoscopic surgery.

Domain	Recommendation	Supporting Evidence	Clinical Implication
**Preoperative**			
ICI exposure history	Document ICI agent, target, number of cycles, last dose, combination therapy, and planned adjuvant treatment.	KEYNOTE-671 [5], RATIONALE-315 [6], DRAGON IV/CAP05 [31], PHERFLOT [30], Tang et al. [20], Ackerman et al. [21]	ICI exposure should be part of routine anesthetic and surgical risk assessment.
Timing of surgery	Surgery may generally proceed after neoadjuvant ICI when the patient is clinically stable and no severe active irAE is present.	KEYNOTE-671 [5], RATIONALE-315 [6], DRAGON IV/CAP05 [31], Tong et al. [14], Cuppens et al. [4], Tang et al. [20]	Prior ICI exposure alone does not appear to justify routine cancellation or prolonged surgical delay; decisions should remain individualized.
Pulmonary assessment	Screen for dyspnea, cough, reduced exercise tolerance, prior pneumonitis, and post-treatment decline in DLCO.	Zhang et al. [26], Tan et al. [28], KEYNOTE-671 [5], RATIONALE-315 [6], Ackerman et al. [21], Sandbank et al. [19].	Thoracic patients may require pulmonary function testing, imaging, and intensified postoperative respiratory monitoring.
Endocrine assessment	Evaluate thyroid and adrenal function when symptoms or laboratory abnormalities suggest endocrine irAEs.	KEYNOTE-522 Japan subgroup [10], RATIONALE-315 [6], VESTIGE [32], Ackerman et al. [21], Sandbank et al. [19]	Unrecognized adrenal insufficiency or thyroid dysfunction may cause perioperative hemodynamic instability.
Cardiac assessment	Investigate chest pain, dyspnea, arrhythmias, unexplained fatigue, or hypotension. Consider ECG and cardiac biomarkers in symptomatic or higher-risk patients and when clinically indicated.	Tang et al. [20], Ackerman et al. [21], Sandbank et al. [19], KEYNOTE-522 Japan subgroup [10]	ICI myocarditis is uncommon but potentially fatal and may mimic perioperative cardiac complications.
Hepatic assessment	Assess liver function before major surgery, particularly after dual ICI therapy or liver-directed treatment.	Lin et al. [34], VESTIGE [32], Ackerman et al. [21], Sandbank et al. [19]	Active immune-mediated hepatitis should prompt multidisciplinary reassessment before elective surgery.
Nutritional assessment	Evaluate nutritional reserve before major gastrointestinal or thoracic surgery.	Cui et al. [35], PHERFLOT [30], DRAGON IV/CAP05 [31]	Poor nutritional status may independently increase postoperative morbidity.
Surgical feasibility	Minimally invasive surgery remains feasible after neoadjuvant immunotherapy in selected patients.	Pan et al. [25], Tong et al. [14], Cui et al. [35], DRAGON IV/CAP05 [31], Zhang et al. [26]	Previous ICI exposure alone should not preclude VATS, robotic, or laparoscopic surgery.
High-risk respiratory timing	Exercise additional caution in thoracic surgery performed shortly after neoadjuvant immunotherapy.	Tan et al. [28], Zhang et al. [26]	Patients may require enhanced pulmonary optimization and postoperative ICU planning.
Multidisciplinary planning	Discuss complex patients in multidisciplinary meetings involving oncology, surgery, anesthesia, and organ-specific specialists.	Ackerman et al. [21], Sandbank et al. [19], Björkström et al. [32], Tang et al. [20]	Multidisciplinary evaluation is recommended for active irAEs, frailty, or major oncologic surgery.
**Intra- and postoperative**			
Hemodynamic management	Anticipate hemodynamic instability, particularly in patients with suspected adrenal insufficiency, myocarditis, dehydration, CRS, or sepsis.	Tang et al. [20], Ciner et al. [22], Ackerman et al. [21], Sandbank et al. [19]	Unexpected vasopressor requirements should prompt consideration of occult irAEs.
Adrenal crisis preparedness	Ensure perioperative corticosteroid availability and stress-dose steroid planning in patients with suspected adrenal insufficiency or hypophysitis.	Ackerman et al. [21], Sandbank et al. [19], KEYNOTE-522 Japan subgroup [10], VESTIGE [32]	Refractory hypotension should prompt early hydrocortisone administration.
Ventilatory strategy	Apply lung-protective ventilation, particularly during thoracic surgery or in patients with previous pneumonitis or impaired DLCO.	Zhang et al. [26], Tan et al. [28], KEYNOTE-671 [5], RATIONALE-315 [6], Ackerman et al. [21]	May reduce postoperative pulmonary complications.
One-lung ventilation	Employ dedicated thoracic anesthetic management with careful one-lung ventilation during lung resections after neoadjuvant immunochemotherapy.	Pan et al. [25], Zhang et al. [26], Tan et al. [28]	Tissue inflammation and fibrosis may increase operative complexity.
Opioid-sparing anesthesia	Opioid-sparing multimodal analgesia may be considered when clinically appropriate, although evidence of an oncological benefit remains insufficient.	Liu et al. [18], Wang et al. [17], Hu et al. [33]	Potential immune-preserving effects have been suggested, although evidence for improved oncologic outcomes remains limited.
Postoperative pain prevention	Anticipate increased postoperative analgesic requirements following neoadjuvant PD-1 blockade.	Wang et al. [6], Pan et al. [25], Liu et al. [18]	Individualized multimodal analgesia is recommended.
Minimally invasive surgery support	Avoid excluding minimally invasive approaches solely because of previous ICI therapy, while remaining prepared for conversion due to fibrosis or adhesions.	Pan et al. [25], Tong et al. [14], Cui et al. [36], Chen et al. [27]	Conversion should not be considered a treatment failure.
Fluid management	Apply individualized goal-directed fluid therapy while avoiding both hypovolemia and fluid overload.	Tang et al. [20], Tan et al. [28], Zhang et al. [26], Ackerman et al. [21]	Appropriate fluid management may reduce pulmonary and renal complications.
Organ-specific monitoring	Escalate intraoperative monitoring when cardiac, pulmonary, endocrine, renal, or hepatic irAEs are suspected.	Tang et al. [20], Ackerman et al. [21], Sandbank et al. [19], Björkström et al. [32]	Postoperative ICU admission or higher-acuity monitoring may be considered based on surgical magnitude, comorbidities, active or prior irAEs, and physiological instability.
CRS differential diagnosis	Include cytokine release syndrome in the differential diagnosis of unexplained intraoperative or early postoperative fever, hypotension, hypoxemia, or multiorgan dysfunction.	Ciner et al. [22], Ackerman et al. [21], Sandbank et al. [19]	CRS may closely resemble postoperative sepsis and requires prompt recognition and treatment.

## Data Availability

No new data were created or analyzed in this study. Data sharing is not applicable to this article.

## Supplementary Materials

The following supporting information can be downloaded at: https://www.mdpi.com/article/10.3390/cancers18162654/s1, Table S1: Complete database search strategies; Table S2: PRISMA 2020 Checklist—Completed.

## Abbreviations

**Abbreviation**	**Full Term**
ACTH	Adrenocorticotropic Hormone
AEGEAN	AEGEAN Phase III Trial
AE	Adverse Event
AKI	Acute Kidney Injury
ALT	Alanine Aminotransferase
AST	Aspartate Aminotransferase
BNP	B-type Natriuretic Peptide
CPS	Combined Positive Score
CRS	Cytokine Release Syndrome
CT	Computed Tomography
CTLA-4	Cytotoxic T-Lymphocyte-Associated Protein 4
CXR	Chest X-ray
DKA	Diabetic Ketoacidosis
DLCO	Diffusing Capacity of the Lung for Carbon Monoxide
DMMR	Deficient DNA Mismatch Repair
ECG	Electrocardiography
eGFR	Estimated Glomerular Filtration Rate
EFS	Event-Free Survival
FDA	Food and Drug Administration
FLOT	Fluorouracil, Leucovorin, Oxaliplatin, and Docetaxel
GI	Gastrointestinal
HER2	Human Epidermal Growth Factor Receptor 2
ICI	Immune Checkpoint Inhibitor
ICU	Intensive Care Unit
IrAE	Immune-related Adverse Event
LAG-3	Lymphocyte Activation Gene-3
LCMC3	Lung Cancer Mutation Consortium 3 Trial
MONEO	Multicenter Perioperative Avelumab Trial
MPR	Major Pathological Response
MSI-H	Microsatellite Instability-High
ADIM	Neoadjuvant Chemoimmunotherapy Trial in Non-Small-Cell Lung Cancer
NEOpredict-Lung	Neoadjuvant Immunotherapy Trial in Lung Cancer
NeoTORCH	NeoTORCH Phase III Trial
NSCLC	Non-Small-Cell Lung Cancer
ORR	Objective Response Rate
OS	Overall Survival
PCR	Pathological Complete Response
PCD-1	Programmed Cell Death Protein 1
PD-L1	Programmed Death-Ligand 1
PFT	Pulmonary Function Test
PRISMA	Preferred Reporting Items for Systematic Reviews and Meta-Analyses
PSM	Propensity Score Matching
M0	Microscopically Margin-Negative Resection
SAKK	Swiss Group for Clinical Cancer Research
TNBC	Triple-Negative Breast Cancer
TSH	Thyroid-Stimulating Hormone
VATS	Video-Assisted Thoracoscopic Surgery
