# Supplementary material for: Perioperative Care of Cancer Patients Treated with Immune Checkpoint Inhibitors: Current Evidence and Clinical Considerations—A Scoping Review
Source: Cancers (Basel). 2026 Aug 17;18(16):2654. doi: 10.3390/cancers18162654 (PMC13510335; doi:10.3390/cancers18162654)
Supplement: Supplementary file 1 [file cancers-18-02654-s001.zip › cancers-4473524-supplementary.pdf]

Supplementary Materials

Manuscript: Perioperative Care of Cancer Patients Treated with Immune Checkpoint Inhibitors: Current Evidence and Clinical Considerations—A Scoping Review

Table S1. Complete Database Search Strategies.

Source	Coverage / limits	Search strategy
PubMed/MEDLINE	1 January 2016–July 2026; English; humans; adults where indexed	(“Immune Checkpoint Inhibitors”[Mesh] OR “immune checkpoint inhibitor*”[Title/Abstract] OR immunotherap*[Title/Abstract] OR PD-1[Title/Abstract] OR PD-L1[Title/Abstract] OR CTLA-4[Title/Abstract] OR nivolumab[Title/Abstract] OR pembrolizumab[Title/Abstract] OR atezolizumab[Title/Abstract] OR durvalumab[Title/Abstract] OR avelumab[Title/Abstract] OR ipilimumab[Title/Abstract]) AND (perioperative[Title/Abstract] OR preoperative[Title/Abstract] OR postoperative[Title/Abstract] OR neoadjuvant[Title/Abstract] OR adjuvant[Title/Abstract] OR surgery[Title/Abstract] OR surgical[Title/Abstract] OR anesthesia[Title/Abstract] OR anaesthesia[Title/Abstract]) AND (cancer[Title/Abstract] OR neoplasm*[Title/Abstract] OR malignan*[Title/Abstract])
Web of Science Core Collection	2016–July 2026; English; document types with sufficient clinical data	TS=(“immune checkpoint inhibitor*” OR immunotherap* OR PD-1 OR PD-L1 OR CTLA-4 OR nivolumab OR pembrolizumab OR atezolizumab OR durvalumab OR avelumab OR ipilimumab) AND TS=(perioperative OR preoperative OR postoperative OR neoadjuvant OR adjuvant OR surgery OR surgical OR anesthesia OR anaesthesia) AND TS=(cancer OR neoplasm* OR malignan*)
Other methods	Reference-list search; same eligibility period	Backward reference checking of included studies and relevant reviews/guidance documents.

Note: The authors should confirm that the database syntax, limits, and final search month above match the searches actually run before resubmission.

Table S2. PRISMA 2020 Checklist—Completed.

Item	Section/topic	PRISMA requirement	Location in manuscript / response
1	Title	Identify the report as a systematic review.	Title and article type identify the work as a literature/scoping review; Methods §2.1 specifies scoping design.
2	Abstract	PRISMA abstract elements.	Structured Abstract: Background, Methods, Results, Conclusions; includes sources, dates, study count, synthesis, and implications.
3	Rationale	Describe rationale in context of existing knowledge.	Introduction, paragraphs 1–11.
4	Objectives	Provide an explicit statement of objectives/questions.	Final paragraph of Introduction.
5	Eligibility criteria	Specify inclusion/exclusion criteria and grouping for synthesis.	Methods §2.2.
6	Information sources	Specify databases and other sources and last search date.	Methods §2.3; PubMed/MEDLINE, Web of Science, reference lists; final search July 2026.
7	Search strategy	Present full strategies, including limits and filters.	Methods §2.3 and Supplementary Table S1 (database-adapted strings to be supplied with submission).
8	Selection process	Specify reviewers, independence, resolution, and automation.	Methods §2.4.
9	Data collection process	Specify charting, checking, author contact, and automation.	Methods §2.5.
10a	Data items—outcomes	List outcomes sought and handling of compatible results.	Methods §2.5.
10b	Data items—other variables	List additional variables and assumptions about missing data.	Methods §2.5.
11	Risk of bias assessment	Specify appraisal methods.	Methods §2.6. Formal risk-of-bias exclusion/certainty assessment was not performed because this was an evidence-mapping scoping review; interpretive appraisal is described.
12	Effect measures	Specify effect measures for each outcome.	Not applicable: no quantitative pairwise synthesis or meta-analysis was performed.
13a	Synthesis—eligibility	Describe decision process for synthesis eligibility.	Methods §§2.2 and 2.7.
13b	Synthesis—data preparation	Describe data preparation methods.	Methods §§2.5 and 2.7; narrative charting and tabulation; missing items recorded as not reported.
13c	Synthesis—display	Describe tabulation/visual display methods.	Methods §2.7; Tables 1–3 and Figure 1.
13d	Synthesis—methods	Describe synthesis methods and rationale.	Methods §2.7; descriptive thematic synthesis; meta-analysis not appropriate because of heterogeneity.
13e	Synthesis—heterogeneity	Describe methods to explore causes of heterogeneity.	Results organized by tumor type, study design, treatment setting, and perioperative domain; no statistical heterogeneity analysis.
13f	Synthesis—sensitivity	Describe sensitivity analyses.	Not applicable: no meta-analysis or pooled estimate.
14	Reporting bias assessment	Describe assessment methods.	Not performed because quantitative synthesis was not undertaken; stated in Methods §2.7 and Limitations.
15	Certainty assessment	Describe certainty assessment.	No formal certainty framework was applied; evidence strength/directness informed interpretation (Methods §2.6).
16a	Study selection—counts	Describe search and selection, ideally with flow diagram.	Results §3.1 and Figure 1.
16b	Excluded studies	Cite apparently eligible excluded reports and explain exclusion.	Figure 1 provides exclusion categories/counts. Individual full-text exclusions were not tabulated because the review mapped heterogeneous evidence; the authors should retain the screening log if requested.
17	Study characteristics	Cite each included study and present characteristics.	Results §3.2 and Table 1 (Appendix A).
18	Risk of bias in studies	Present risk-of-bias assessments.	Not applicable as a formal outcome; rationale in Methods §2.6.
19	Results of individual studies	Present summary data/results for each study.	Results §§3.2–3.6 and Table 1.
20a	Results of syntheses—summary	Summarize contributing studies and risk of bias.	Results §§3.2–3.6; study design and limitations summarized narratively.
20b	Statistical syntheses	Present meta-analysis results and heterogeneity.	Not applicable: no meta-analysis.
20c	Investigations of heterogeneity	Present subgroup/meta-regression results.	Not applicable statistically; narrative variation by tumor type and clinical context is reported.
20d	Sensitivity analyses	Present sensitivity analyses.	Not applicable: no meta-analysis.

21	Reporting biases	Present risk of bias due to missing results.	No formal assessment; publication and language bias acknowledged in Limitations.
22	Certainty of evidence	Present certainty assessments.	No formal certainty grading; evidence limitations and directness discussed in Discussion and Limitations.
23a	Discussion—interpretation	Provide general interpretation in context.	Discussion, opening and thematic paragraphs.
23b	Discussion—evidence limitations	Discuss limitations of included evidence.	Discussion and Limitations.
23c	Discussion—review limitations	Discuss limitations of review processes.	Limitations.
23d	Discussion—implications	Discuss implications for practice and research.	Discussion and Conclusions.
24a	Registration	Provide register name and number or state not registered.	Methods §2.1 and Registration and Protocol statement: PROSPERO CRD420261451278.
24b	Protocol access	Indicate where protocol can be accessed.	PROSPERO record URL in Registration and Protocol statement.
24c	Amendments	Describe amendments.	Registration and Protocol statement: no amendments affecting eligibility or synthesis methods.
25	Support	Describe financial/non-financial support and funder role.	Funding statement. No additional non-financial support reported.
26	Competing interests	Declare competing interests.	Conflicts of Interest statement.
27	Availability of data/materials	Report availability of forms, extracted data, code, and materials.	Evidence tables are included in the manuscript. No analytic code was generated because no meta-analysis was performed; screening and charting records are available from the corresponding author on reasonable request.

Note: For a scoping review, PRISMA-ScR is the design-specific extension; the full PRISMA 2020 checklist above has nevertheless been completed in response to the editorial request. Items related exclusively to meta-analysis are marked not applicable with reasons.
